# The History, Diagnosis, and Treatment of Edematous Laryngitis

**Published:** 1850-03

**Authors:** Elisha Bartlett

**Affiliations:** Professor of the Theory and Practice of Medicine in the University of Louisville


ART. II.—*The History, Diagnosis, and Treatment of Edematous Laryngitis.* By ELISHA BARTLETT, M. D., Professor of the Theory and Practice of Medicine in the University of Louisville.

### ARTICLE I.—INTRODUCTORY.

I have been induced to prepare this memoir, principally by the following considerations. The disease, of which it gives an account, runs its course with great rapidity, and is attended with extreme danger; terminating, nearly always, in death, unless arrested by timely and active interference. There is good ground for believing that this disease is of more frequent occurrence than has generally been supposed. There is no full and complete history of it, generally accessible to American physicians. There is no impropriety in saying that no one of the popular and standard works on practical medicine, either English or American, usually found in the hands of our medical men, and relied upon as guides and authorities, furnishes such knowledge of this disease as is essential to its successful treatment. This knowledge ought at all times to be ready for use and application. The necessity for this use and application, whenever it occurs, is immediate, urgent, and pressing; it admits of no delay; there is no time for deliberate inquiry and consultation. The physician, in many, perhaps in most cases, is obliged to act upon his own single responsibility; it is his duty to be fully prepared, in advance of the emergency, to act promptly and aright.

The sources from which the materials, made use of in the composition of this paper, have been derived, will be indicated in their appropriate places. I have nothing of my own to add to these materials, except the vivid and painful recollection of two fatal cases of the disease, occurring in private practice, both of which, with our present means and knowledge, might probably have been saved.*

### ARTICLE II.—Lesions.

Sec. 1. *Preliminary.*—The pathological anatomy of edematous laryngitis constitutes the necessary and indispensable foundation of everything like an adequate and complete knowledge of the disease. Its symptoms, its pathological relations, and its treatment, can be rendered intelligible only by a reference to its lesions. If there were still to be found, in the ranks of our profession, any individuals of that class who are disposed to depreciate the value and to underrate the importance of pathological anatomy, they could hardly be referred to any more striking or emphatic refutation of their prejudices than that which is furnished by this disease. All its phenomena, all its relations, and its entire treatment, are bound up in its appreciable and obvious anatomical lesions, and are directly dependant upon them.

Sec. 2. *Infiltration.*—Edematous laryngitis consists, in its essential anatomical element, in an infiltration and a consequent tumefaction of the lax submucous cellular tissue, upon the anterior surface of the epiglottis, and around the edges of the glottis. Bayle's original description of the diseased parts is in the following words: "In all cases, the edges of the glottis are swollen, thickened, white and trembling; they form a swelling more or less prominent, fully infiltrated with serum, which is pressed out from the cellular tissue with great difficulty, even after free incisions have been made in it. * * * The edges of the infiltrated and swollen glottis are dis-

* Different appellations have been applied to this disease. Bayle, its first historian, called it "*Edema of the Glottis*," or "*Edematous Laryngeal Angina.* Curveilhier calls it "*Submucous Laryngitis.*" Valleix, Grisolle, and many others apply to it indifferently the terms: "*Edema of the Glottis*," and "*Edematous Laryngitis.*"

posed in such a manner, that every impulsion which comes from the pharynx turns them down upon the opening of the glottis, which is thus more or less obstructed,—while an opposite impulsion, from the trachea, throws these moveable folds upwards and outwards, upon the sides of the opening of the glottis, rendering the orifice comparatively or entirely free. * * * * The epiglottis is rarely intact; it is often very much swollen upon its edges."*

I have thought it best to add to the foregoing, even at the cost of some repetition, Hasse's minute and excellent description of the lesions. "The inflammation," he says, "assails the mucous membrane above the glottis, and particularly those of its folds which unite the epiglottis on the one hand with the root of the tongue and the arches of the palate, on the other, with the larynx, especially laterally, in the direction of the arytenoid cartilages—these folds being, as is well known, very lax and moveable, and susceptible of great extension. * * * Inflammation of the epiglottis exhibits, after death, the whole of the mucous membrane between the root of the tongue and the glottis uniformly tumefied, so that the outlines of the epiglottis and arytenoid cartilages, together with the numerous folds and recesses in the vicinity of those parts have became effaced. This is the result of inflammatory effusion into the interspaces of the loose cellular texture, subjacent to the mucous membrane. In proportion to the intensity and duration of the inflammatory process, this exudation is sometimes of a purely serous and liquid nature, so as to flow away upon incision; sometimes blended with coagulable materials, and jelly-like; sometimes, again, mingled in various proportions with pus; sometimes wholly purulent. Hence the tumor, which is always soft, lax and tremulous, like jelly, varies

---

*Dic. des Sci. Med., vol. xviii. p. 510.

greatly in color, being of a pale, or of a reddish yellow, sometimes of a dingy yellow or grayish white, and more or less opaque,—but, for the most part, superficially dotted with red. The swelling being dependant upon infiltration of the submucous cellular texture, cannot extend to the inferior surface of the epiglottis, because, there, no layer of cellular tissue exists ; hence, the epiglottis has the aspect of having both its lateral edges bent over towards its nether surface, so as to form a narrow perpendicular groove, which is sometimes almost covered by the overhanging tumor. Neither does the swelling extend to the vocal ligaments—so that the term "*edema glottidis,*" is not strictly correct—but the tumor hanging down on each side, and in size often exceeding a pigeon's egg, overlays the glottis in such wise as to leave but a narrow opening towards the posterior part which allows the column of air to pass out during expiration, but is closed up by any attempt at inspiration. In no example recorded, did the edematous infiltration beneath the mucous membrane show itself in any marked degree beyond the glottis."*

It will have been noticed in the foregoing descriptions, that while Bayle speaks only of a purely serous infiltration, Hasse states the infiltration to be sometimes serous, sometimes sero-purulent, and sometimes wholly purulent. There can be no doubt that the latter statement is the true one. Valleix says that, in more than three quarters of the cases, collected by himself, the infiltration was more or less mingled with pus. According to Valleix, in a few cases, the infiltration extends to the vocal cords.†

SEC. 3. *State of the Mucous Membrane.*—In a small number of cases, the infiltration above described constitutes the only alteration. The mucous membrane, and

---

* Hasse's Path. Anat., pp. 258—260.
† Mem. de l'Acad. Roy. de Med., vol. xi., p. 107.

the parts in the neighborhood of the glottis are quite free from any obvious disease. This, however, is not generally the case. In most instances, there are traces of inflammation in the mucous membrane, or some other lesion in or about the glottis. Valleix found the mucous membrane red in one-third of his cases, ulcerated in about one-sixth, in others detached from the subjacent tissues, and so on.

Hasse says: "The mucous membrane of the affected parts is always variously changed. Its surface is rough; its epithelium partly thrown off in single scales and tufts, or raised by serous fluid; it exhibits, here and there, blood specks, or scattered bright red vascular streaks; it is, moreover, easily separable, and, when separated, friable. The neighboring muscles, especially the arytenoid, are sometimes unaltered, at other times saturated in like manner with the effused fluid, in which case they have become blanched, or undergone yellowish or grayish softening.*

Another common lesion consists of ossification, caries, or necrosis, of the laryngeal cartilages, especially the cricoid. Valleix found some lesion of the cartilages in more than half his cases. In a few instances, ulceration or abscesses have been found in the pharynx. The foregoing alterations are frequently found, more or less of them, together.

### ARTICLE III.—Symptoms.

Sec. 1. *Mode of Access.*—Edema of the glottis sometimes commences suddenly, with a formal and violent paroxysm of suffocative dyspnea. This, however, is a rare occurrence. Valleix says that of forty cases, the histories of which he had examined, two only commenced in this manner.† The access is generally somewhat

* Hasse's Path. Anat., p. 260.

† Mem. de l'Acad. Roy. de Med., vol. xi., p. 136.

gradual, although more or less rapid,—the disease usually reaching its stage of full development, in from a few hours to two or three days. The principal phenomena of this period are, a feeling of uneasiness in the larynx ; an irresistible inclination to swallow, occasioned by the sensation of a foreign body in the pharynx, or upper part of the esophagus ; efforts, by hawking, to clear the larynx and fauces ; some difficulty of respiration ; and slight hoarseness. The hand of the patient may be frequently carried to the seat of uneasiness in the larynx.*

SEC. 2. *Pain in the Larynx.*—This pain, either spontaneous, or excited by pressure, is one of the earliest and also one of the most constant symptoms of the disease. It is mentioned as present, in some form or degree, in 32 of 40 cases, by Valleix; and in only one of the remaining eight is its absence formally stated. The pain is not often at all urgent, and in most cases does not attract the notice of the patient until his attention is called to it. In many instances, instead of positive pain, there is a pricking sensation in the larynx, some uneasiness on pressure, the feeling of a foreign body in the throat, or a sense of strangulation.†

SEC. 3. *Pain in the Pharynx.—Difficulty of Deglutition.*—In a certain proportion of cases there is pain in the pharynx, independent of the act of swallowing. Pain and difficulty of swallowing are present in a large majority of cases; in many instances they are extreme, and sometimes the act of swallowing becomes impossible. In three of Valleix's cases, drinks were returned by the nose.‡

It results, then, from the foregoing statements that pain about the throat, in the larynx or pharynx, or both, varying in kind and degree, spontaneous, or excited by pressure or motion, is an almost invariable accompaniment of

* Dic. des Sci. Med., vol. xviii., p. 508.
†Mem. de l'Acad. Roy. de Med., vol. xi., p. 136.
‡Mem. de l'Acad. Roy. de Med., vol. xi., p. 138.

the disease. It was noticed in 38 of Valleix's 40 cases.*

Sec. 4. *Cough and Expectoration.*—Cough is neither an urgent nor a constant sympton. Bayle merely says that, in the progress of the disease there supervenes in some cases a slight and occasional cough.† It is mentioned in only 14 of Valleix's 40 cases, and in the most of these it was slight. In some cases the cough is dry; in others there is more or less expectoration, varying in character in different patients, and in no way distinctive of the disease. Both the foregoing symptoms are of secondary importance.

Sec. 5. *Voice.*—One of the most constant symptoms of the disease consists in an alteration of the voice. This becomes rough and hoarse, then more and more muffled, and finally either nearly or altogether extinct. In only one of Valleix's cases had it the croupal character.‡

Sec. 6. *Respiration.*—The capital and most characteristic symptom of edema of the glottis is the difficulty and embarrassment of the respiration. It is an invariable attendant upon the disease, and by the extreme and intolerable distress which it occasions, it concentrates and absorbs almost the whole attention both of the physician and the patient. It is necessary to describe this symptom in detail.

It has already been stated that, in a few cases, edema of the glottis may commence with sudden and extreme dyspnea. Generally, however, the difficulty of respiration, showing itself at a pretty early period of the disease, is at first only slight or moderate in degree; it subsequently goes on, more or less steadily and regularly, increasing in severity until it reaches its highest point of intensity.

There is a very striking peculiarity of the dyspnea, first noticed by Bayle, and since by many observers. This

---

*Mem. de l'Acad. Roy. de Med., vol. xi., p. 141.

†Dic. des Sci. Med., vol. xviii., p. 508.

‡Mem. de l'Acad. Roy. de Med., vol. xi., p. 146.

peculiarity consists in the great difference in the difficulty of *expiration* and of *inspiration*. The latter is very much more difficult and laborious than the former. Bayle states, without any qualification, that while inspiration is very difficult, noisy, and hissing, expiration is always performed freely and easily. This statement requires some qualification. It is generally but not absolutely correct. In a large proportion of cases, the expiration does remain quite free and unembarrassed; but not in all. Valleix says that in five of eighteen cases the expiration was more or less difficult; but that in these cases the expiration was less difficult than the inspiration.

The difficult respiration is persistent and continued, but in most cases it is marked at intervals by paroxysms of extreme suffocative dyspnea. The following is Bayle's vivid and truthful description of these frightful attacks: "When the paroxysm is violent," he says, "the patient, upright upon his seat, experiences an extreme difficulty in breathing; his shoulders are forcibly elevated, all his chest is in motion, inspiration is very laborious and noisy, while expiration remains free and facile; he is threatened with imminent suffocation; the face is pale and pinched, or flushed and swollen, with a fearful or wandering expression; some patients demand earnestly and imploringly that the larynx should be opened; others seek a cutting instrument with which to rid themselves of the substance that suffocates them; in many cases there are moments of fury during which patients are tempted to put a violent end at once to their agony and life; they beat the bed with their hands, are in constant and violent agitation, and give utterance to cries of terror and despair. In these extreme paroxysms, and even in those that are much less violent, the pulse becomes unequal, irregular, and sometimes intermittent. When the fit has subsided, the breathing may become comparatively easy, but the pulse continues feeble and irregular. Not unfrequently, after a very short respite, another paroxysm or a series of paroxysms, carries off the patient; but more commonly death takes place during the comparative calm that follows the fit."*

Sec. 7. *State of Pharynx.*—In most of the cases that have been reported no mention is made of the appearance of the pharynx, and its examination seems to have been generally neglected. Valleix says, however, that of thirteen cases, in which the fauces were examined, there was only one which did not exhibit some appreciable lesion. In all the others there was manifest redness, more or less extensive, of the mucous membrane, with or without tumefaction; in two instances the semi-transparence of the swollen tissue showed that the tumefaction was owing to serous infiltration.† In four of the five cases reported by Dr. Buck, there was inflammation, more or less extensive, in and about the fauces.‡

Sec. 8. *Signs derived from Touch.*—The edematous swelling of the lax sub-mucous cellular tissue, upon the upper side of the epiglottis, and along the edges of the glottis, constituting anatomically the disease under consideration, may be felt and recognized by the touch. This very important and positive means of diagnosis was first discovered by Tuilier; it was announced in a thesis presented to the Faculty of Medicine of Paris in 1815. Valleix and Dr. Buck, and there can be no higher authorities, add the testimony of their own repeated experience to the original statement of Tuilier. In Dr. Buck's five cases, before scarification was resorted to, the existence of the edema of the epiglottis was positively ascertained by the touch. Dr. Buck speaks particularly of the swelling of the *epiglottis* only, but in the third case, he says,

*Dic. des Sci. Med., vol. xviii, p. 509.
†Mem. de l'Acad. Roy. de Med., vol. xi., p. 149.
‡Trans. Amer. Med. Asso., vol. i., pp. 137, 144.

"while the end of the finger was pressing the epiglottis against the base of the tongue, the soft pulpy swollen edges of the glottis were felt rising up against it."* Valleix says, that of twelve cases, in which the exploration by touch was methodically made, the edematous swelling of the edges of the glottis was felt in eight. The swollen epiglottis, says **Dr. Buck**, conveys to the touch the sensation of a soft pulpy body, easily recognized and distinguished from the stiff rigid swelling of these parts in membranous laryngitis.

Sec. 9. *Physiognomy.*—The state of the face and its expression are such as might be looked for in a disease consisting essentially in a slow strangulation. The face is sometimes flushed, congested, and swollen; sometimes pale and sunken; the lips are livid, the eyes wild and staring; and the varying and commingled phases and degrees of bodily and mental agony, the dreadful sense of suffocation, the intense longing for fresh air, the consciousness of near and imminent danger, are vividly impressed upon the physiognomy.

Sec. 10. *General Symptoms.*—These are neither constant nor important. In some cases there is more or less febrile excitement; in others it is altogether wanting. The pulse is generally accelerated, becoming very rapid, feeble, and fluttering with the progress of the disease. The restlessness and agitation of the patient, at least before the gradual asphyxia is much advanced, are in proportion to the degree of dyspnea. There is sometimes thirst; the appetite may be diminished or lost. If there is any disturbance of the stomach or bowels it is quite accidental.

### ARTICLE IV.—Causes.

Sec. 1. *Preliminary.*—Edematous laryngitis does sometimes occur spontaneously in persons at the time in good

*Trans. Amer. Med. Asso., vol. i., p. 139.

health, not suffering at the time with any general or local disease, and without any appreciable determining cause. This, however, is not common. It happened only three or four times in Valleix's 40 cases. In most instances the disease is connected with some general morbid condition or with some local affection.

SEC. 2. *Local Disease in and about the Glottis.*—The frequency of this complication has already been stated in describing the anatomical lesions of the disease. These primary affections, acting as determining causes of the edematous laryngitis are thus enumerated by Valleix: 1. Simple inflammation of the laryngo-pharyngean mucous membrane. 2. Ulceration, acute or chronic, of the larynx, and sometimes of the pharynx. 3. Simple abscess of the pharynx, and sometimes of the larynx. 4. Alterations, more or less profound, of the laryngeal cartilages, with submucous abscesses, or disease of the mucous membrane. 5. In rare instances, inflammation of an organ more remote, such as the tongue.*

SEC. 3. *Convalescence from Fevers.*—The frequent occurrence of edematous laryngitis during convalescence from low fevers was noticed by Bayle, the first historian of the disease. As to the primitive form of this angina, he very truly says, it comes on most frequently during convalesence from febrile diseases of a grave character, such as adynamic or ataxic fevers.† There was an extraordinary frequency of the disease in the New York Hospital, between the months of December, 1847, and February, 1848. During this period, says Dr. Buck, the season was remarkably rainy and wet, accompanied with very little snow, and characterized by the prevalence of erysipelas and typhus fever, as well as an asthenic type in other diseases, both in and out of the hospital.‡

---

* Mem. de l'Acad. Roy. de Med., vol. xi., p. 120.
† Dic. des Sci. Med., vol. xviii., p. 508.
‡ Trans. Amer. Med. Asso., vol. i., p. 135.

The following is a tabular view of the circumstances under which the forty cases, analyzed by Valleix, occured:

| | |
|---|---|
| During convalescence from grave fevers, - | 10 |
| During convalescence from pneumonia, - | 4 |
| In the course of erysipelas, - - - | 1 |
| After scarlet fever, - - - - | 1 |
| After lithotomy, - - - - - - | 1 |
| During treatment of fracture, with fever, - | 1 |
| During convalescence from cerebral congestion, | 1 |
| In the course of bronchitis, - - - | 1 |
| In the course of hypertrophy of the heart, - | 1 |
| In the course of elephantiasis, - - | 1 |
| In the course of laryngeal phthisis, - - | 9 |
| In the course of cancer of the larynx, - | 1 |
| With syphilis, - - - - - | 2 |
| During good health, - - - - | 4 |
| State of health not mentioned, - - | 2 |

Sec. 4. *Age; Sex; Season.*—The following table shows the ages in 36 cases cited by Valleix:

| | |
|---|---|
| Under 10 years, - - - - | 2 |
| From 10 to 20 years, - - - | 5 |
| From 20 to 30 " - - - - | 8 |
| From 30 to 40 " - - - | 4 |
| From 40 to 50 " - - - - | 8 |
| From 50 to 60 " - - - - | 5 |
| From 60 to 70 " - - - - | 3 |
| At 71, - - - - - - | 1* |

The disease is much more common in the male than in the female sex. Of Valleix's 40 cases, 29 occurred amongst males, and 11 amongst females.

It does not appear that season or weather has any very marked influence in the production of the disease. Of 39 cases, mentioned by Valleix, 21 occurred between Oc-

* Mem. de l'Acad. Roy. de Med., vol. xi., p. 121.

tober and March, and 18 between April and September.*

### ARTICLE V.—Varieties and Forms.

Edematous laryngitis, as it has been described in the foregoing pages, has been divided, especially by Cruveilhier, into *primitive*, and *secondary*, and also into *acute* and *chronic*. The grounds of this division are obvious enough and it is only necessary to remark here that the strictly chronic form of the disease must be of extremely rare occurrence, if, indeed, it is ever met with.

But, further than this, Cruveilhier divides edematous laryngitis, or, as he calls it, *submucous* laryngitis, into two varieties;—first, the *supra-glottic*, the ordinary form already described; and second, the *infra-glottic*, in which the inflammation is seated below the glottis. M. Cruveilhier reports five cases of this form of disease. They were accompanied by necrosis of the cricoid cartilage, and by one or more abscesses, in immediate contact with it. In two instances, the abscesses opened into the esophagus. M. Cruveilhier regards the necrosis of the cartilage as the result of the inflammation in its immediate neighborhood.† It seems to me hardly proper to call this inflammation of the cellular tissue of the larynx, about the cricoid cartilage, followed by abscesses, and not accompanied by any infiltration of the submucous cellular tissue of the glottis, a form or variety of the disease under consideration.

Cases occurring in connexion with erysipelas have been elevated by some writers into a distinct form of the disease.

---

* Mem. de l'Acad. Roy. de Med., vol. xi., p. 123.

†Dic. de Med. et de Chir. Prat., vol xi., pp. 41—45.

### ARTICLE VI.—March; Duration; Termination.

The usual progress of the disease is contained in the history of the symptoms, already given. This progress, or march, may, however, vary considerably in different cases. It would seem that in cases depending upon simple inflammation, the march of the disease is usually regular and gradual; while in those which supervene upon some chronic lesion, the access is not unfrequently announced by a sudden paroxysm of suffocative dyspnea. According to Valleix, the disease, when fully established, marches with a rapidity in proportion to the intensity of the inflammation.

The most striking feature in the march of the disease, consists in the violent suffocative paroxyms already described. These are present in a large proportion of cases. Bayle says they usually make their attack during sleep. They generally increase in frequency and violence with the progress of the disease. They occur more frequently during the night than during the day. The degree of relief in the intervals of comparative calm between the paroxysms, differs very much in different cases. Sometimes, mostly in the early period of the disease, it is very considerable; in other cases, and usually late in the disease, it is very slight.

The duration of the disease varies from only a few hours to two or three weeks. In a large majority of cases, however, unless the patients are relieved by a surgical operation, the disease is not prolonged beyond four or five days. This duration, according to Valleix, is generally longer in cases supervening upon chronic disease of the larynx, than in those that are primitive, or simply inflammatory.*

Death may take place during one of the suffocative

* Mem. de l'Acad. Roy. de Med., vol. xi., p. 162.

paroxysms, or in the period of comparative calm that follows. The latter is more frequent than the former—a fact that was noticed by Bayle. Of the cases, constituting the basis of Valleix's memoir, three only terminated in the midst of a violent paroxysm; in seven, death took place during a period of comparative calm; but in all the others, constituting a considerable majority of the cases, the fatal termination took place, neither in the midst of a distinct paroxysm, nor during a period of well marked repose, but in a state of steadily progressive asphyxia, depending upon the persistent and gradually augmenting difficulty of respiration. In only one instance was death quite sudden.*

### ARTICLE VII.—Mortality and Prognosis.

Edematous laryngitis, unless relieved by a surgical operation, generally terminates in death. Bayle, when his first memoir upon the disease was written, in 1808, had seen seventeen cases—all of which were fatal. His subsequent experience did not induce him essentially to change his prognosis. Of the forty cases, collected by Valleix, nine terminated in recovery. This, it seems to me, is an unusually large proportion. It appears probable that the primitive form of the disease, occurring in persons otherwise in good health, is somewhat more susceptible of cure, than that which is connected with other lesions of the larynx, or supervenes in the course of some other disease.†

### ARTICLE VIII.—Diagnosis.

A well marked case of edematous laryngiti , attended by its ordinary phenomena, is not very likely to be confounded with any other disease. Pain or uneasiness

*Mem. de l'Acad. Roy. de Med., vol. xi., p. 158.
†Mem. de l'Acad. Roy. de Med., vol. xi, p. 173.

about the larynx; the sensation of a foreign body in the throat; accompanied or followed by more or less difficulty of swallowing; laborious, difficult, and noisy inspiration, the expiration often remaining entirely or comparatively free; agitation; occasional paroxysms of suffocative dyspnea of extreme violence; and sooner or later signs of gradually increasing asphyxia,—constitute a combination of phenomena, very characteristic of this disease, and certainly not often found in any other. For an absolutely positive diagnosis, we must rely upon the distinctive physical sign dependent on the touch. Grisolle doubts the value of this last means of diagnosis; he says that in two or three instances, he attempted, but unsuccessfully, to reach with his finger the seat of disease; that the spasmodic contractions of the upper orifice of the larynx, and the efforts at vomiting obliged him to desist, and that the same thing had happened to Chomel, Blache, and others.* The testimony of Valleix and Dr. Buck, as well as of many others, leaves no doubt, however, of the practicability of this exploration.

Edematous laryngitis may sometimes be confounded with exudative inflammation, or true croup. In the latter, however, a careful examination of the fauces will generally exhibit patches of the effused fibrin; and any uncertainty that might still exist would be removed by the touch. Beside certain differences in the voice, cough, and breathing, which are, not always, but commonly, present—it will be remembered also that edematous laryngitis is a disease of adult life, while true croup almost always occurs in childhood. I do not know how frequent simple exudative laryngitis, or true croup, may be in the adult; but I suppose many of the cases which have been regarded and reputed as such, have been cases of edematous laryngitis. The latter disease, independent of the

* Traitè de Path. Int., vol i., p. 317.

class of cases occasioned by scalding water, does occur also in children, but it seems to be very unfrequent. It is a striking, and as it appears to me, a singular pathological fact, that it should occur so rarely, if it does at all, in connexion with membranous laryngitis.

It is said, further, that edematous laryngitis has been or may be confounded with purulent infiltration or absceses in and about the larynx and pharynx; aneurism of the aorta pressing upon the trachea; a foreign body in the air passages; asthma, and so on. Such difficulties may sometimes be met with, but with our present means of diagnostic investigation, and a careful application of them, they will be so rare and exceptional as to be of but little importance.

### ARTICLE IX.—Theory.

I do not think there can be any reasonable doubt as to the nature of this disease. Two principal opinions have been adopted in regard to it. One of these is that the disease is a simple dropsy, and that when inflammation is present this is accidental. This was the doctrine of Bayle. He regarded the disease as essentially unlike laryngitis. The other opinion is, that the disease is inflammatory, and that the cellular infiltration is the result of inflammatory action. I think this view is the true one. It is very ably, and I think quite conclusively argued and illustrated by Valleix. The principal reasons for it are these. In many cases, there is pus in the cellular tissue; these cases must at least have had an inflammatory element in them. In many cases, the infiltration, even if wholly serous, is evidently connected with and dependant upon some local inflammation in its immediate vicinity. The fact seems to be, in these cases, that a slight degree of inflammatory irritation spreads from the original inflammation to the loose cellular tissue about the glottis, and thus gives rise to the effusion. I do not say that there cannot be, or that there is not, occasionally, an instance of pure, non-inflammatory edema of the glottis. Such an assertion would not be in accordance with our rigorous and positive philosophy. I only mean to say, that in very many cases, there is conclusive proof of the inflammatory character of the disease, and that in those where this proof is wanting, the fairest and most rational interpretation of all the phenomena is in no way inconsistent with this doctrine. No fact in pathology is better established, than that many inflammations, especially those of recent occurrence, and of moderate or slight severity, like that under consideration, leave no traces or results that are appreciable after death. The conclusion to which I come, then, is this, that edema of the glottis is the result of an inflammatory action, more or less violent, spreading in many cases from an original inflammatory centre in the neighborhood of the glottis, or extending from the mucous membrane covering the infiltrated tissue.

M. Fleury has controverted this view, and insists very earnestly on the purely dropsical and non-inflammatory nature of the disease. I have not seen his paper. It is quoted by Valleix.*

### ARTICLE X.—TREATMENT.

SEC. 1. *Medical Means.*—The simply medical treatment of this disease, however prompt, energetic, and judicious, is generally unsuccessful. The whole history of the disease bears witness to the truth of this. Still, it is none the less true, that cases are sometimes saved, apparently by treatment. Cruveilhier is very confident that he has arrested the progress of the disease in many instances. The means, generally used and principally relied upon, are bleeding, general and local; emetics; mer-

---

* Mem. de l'Acad. Roy. de Med., vol. xi., p. 129.

curials; and blisters to the throat. I do not know that there is any objection to the proper and timely use of these means; only let it be remembered that they are much more likely to fail than to succeed, and that they are not to be depended upon.

SEC. II. *Scarification of the edematous parts.*—Dr. Marshall Hall published, in 1821, in the Medico-Chirurgical Transactions, an account of four cases of inflammation of the throat, occurring in children and occasioned by attempting to drink scalding water from the mouth of a teakettle. In speaking of the treatment, he says: "If the suffocation were imminent, I should not hesitate to propose laryngotomy, or tracheotomy, and the former would appear to reach below the seat of the affection.—*But I now regret that I did not propose scarification of the epiglottis and glottis, so as to evacuate the blisters.*" This most important and sagacious suggestion seems to have attracted no attention.

M. Lisfranc published a paper on the disease in the Journal Generale, for 1825, in which he gives a history of five cases treated by scarifying the edematous parts. I have not been able to procure this article, and I copy the following account of the operation, written by Valleix, from Dr. Buck's paper: "M. Lisfranc first conceived the idea of evacuating by means of incisions more or less numerous, the serous or sero-purulent fluid engorging the submucous tissue of the larynx. This surgeon cites five cases in which this operation was followed by an immediate change, and subsequently by a complete cure. In a sixth case, several similar operations, at variable intervals, acted only as palliatives. Extensive lesions of the larynx existed, which at length caused the death of the patient.

"The following is M. Lisfranc's mode of scarifying the larynx: Take a long, narrow-bladed, slightly-curved bistoury, in a stiff handle, protected with a strip of linen to within half an inch of the point. Let the patient open his mouth wide, and have the jaws kept apart by means of a cork placed far back between the molar teeth, one end of the cork being held by an assistant. The patient being placed in front of the operator with his head supported against the breast of an assistant, pass the index and middle finger of the left hand into the mouth, till they reach the swollen edges of the larynx, glide the bistoury flatwise upon the finger, holding it as you would a pen. On reaching the larynx, direct the edge forward and upward, then after having elevated the handle, depress it gradually, at the same time pressing gently upon the point. At first a few punctures only should be made, as by the aid of pressure two or three small incisions are sufficient. They may easily be multiplied in the same way if judged necessary.

"These scarifications produce a flow of the infiltrated matter and sometimes a slight oozing of blood, which effects a salutary disgorgement. The cough, excited by a few drops of serum falling into the larynx, contributes much to diminish the swelling. The immediate beneficial results of these scarifications might be partially defeated, by their occasioning more or less inflammation of the larynx and surrounding parts. In such a case recourse must be had to general or local bleeding, which would soon disperse this traumatic inflammation."*

This most interesting and important announcement of the celebrated French surgeon seems to have attracted but little favorable notice. It is difficult to understand the causes of this, unless they are to be found, in part at least, in the unfriendly personal relations so notoriously existing, for many years, between M. Lisfranc and most of his distinguished contemporaries, and the ferocious

*Dr. Buck's paper on Edematous Laryngitis. Trans. Amer. Med. Asso., vol i., p. 150.

and violent hostility which he so constantly exhibited towards them. Some ten years after the publication of M. Lisfranc's memoir, M. Cruveilhier says, certainly with a deliberate rudeness—a much harsher term would be more appropriate—as inexcusable as it is unaccountable: "Finally, M. Lisfranc has proposed to make scarifications upon the infiltrated parts. *I doubt whether this littlitte operation has ever been performed!*"* M. Valleix, some twenty years after the appearance of Lisfranc's memoir, remarks that the operation proposed and practiced by him, *ought to be taken into serious consideration.* He thus enumerates and answers the principal objections that had then been made to it. First, it was alleged that in a large number of cases, the edematous swelling could not be recognized by the touch. The answer to this is, that if the exploration is properly and skilfully made, the edematous tumors *can be, and are*, in most cases, recognized; and that where they are not felt, the operation is not to be attempted. The second objection consisted in the alleged difficulty of the operation. "But," says M. Valleix, "this objection is not a serious one, since the difficulty was overcome so successfully in the cases of M. Lisfranc." Finally, it was said, that admitting the possibility of making the scarifications, only a slight amelioration could be obtained, since, according to the observations of Bayle, the infiltrated fluid escapes very slowly, even when numerous incisions have been made. To this objection, it is sufficient to reply, that in Lisfranc's cases, the relief was immediate, and consequently the escape of fluid must have been sufficiently free.†

It appears, also, that M. Marjolin, on some occasion, though at what time I am unable to say, lacerated the edematous edges of the larynx with a piece of althea root,

---

*Dic. de Med. et de Chir. Prat., vol. xi., p. 41.
†Mem. de l'Acad. Roy. de Med., vol xi., p. 182.

and that the patient recovered. Acting on this hint, M. Legroux, I suppose in 1839, proposed lacerating the edematous tumors with the finger nail, sharpened and roughened for this purpose. In two cases, M. Legroux says, he saved his patients by this simple means. In regard to all the foregoing direct surgical measures, M. Valleix very sensibly and properly says: "It would be very wrong to contemn or neglect them, for it is certain that many times the lives of patients have been saved by them."*

Mr. Frederick Ryland, the author of an elaborate and in many respects very valuable monograph upon diseases of the larynx and trachea, and which has been pretty extensively circulated in this country, by its reprint in Dunglison's American Medical Library, writing as late as 1837, disposes of this most grave and important matter in a very summary and quite a different manner. After alluding first to the plan of M. Tuilier, of making pressure from time to time, by means of the finger, upon the distended lips of the glottis, in order to promote the ab sorption of the effused serum, and then to Lisfranc's operation, he says: "But both plans are fantastic, very difficult, if not impossible of accomplishment, and more likely to increase than diminish the existing mischief."†

Mr. Hodgkin, in 1840, says: "Were it possible to effect a slight scarification of the edematous parts, not only immediate relief but permanent advantage would probably be derived from it."‡

Such are the principal facts, as far as I have been able to ascertain them, in the history of this operation, up to the years of 1847 and 1848. At that time, a most important addition was made to our experience, and a new and fresh interest given to the subject by the observations

*Mem. de l'Acad. Roy. de Med., vol. xi., p. 184.

†Ryland on Dis. and Inj. of Lar. and Trachea.

‡Lect. on Morb. Anat., vol. ii, p. 51.

of Dr. Gurdon Buck, of New York, one of the surgeons of the New York Hospital. It has already been stated that between the months of December 1847, and February 1848, there occurred in the hospital no less than seven cases of this disease. Dr. Buck, in his own words, more than a year previous to the occurrence of the first of these cases, and without any knowledge at that time of any similar method of cure having been practiced or proposed by others, was led to the conviction that scarifications of the edematous edges of the glottis, as well as of the epiglottis, might be employed as an effectual means of relief in this formidable disease.

The following is Dr. Buck's description of his mode of operating:—

The patient being seated on a chair, with his head thrown back, and supported by an assistant, he is directed to keep his mouth as wide open as possible; and if there be any difficulty in this respect, a piece of wood an inch and a quarter in width, and half an inch in thickness, is to be placed edgewise between the molar teeth of the left side. The fore-finger of the left hand is then to be introduced at the right angle of the mouth, and passed down over the tongue till it encounters the epiglottis.

But little difficulty is generally experienced in carrying the end of the finger above and behind the epiglottis so as to overlap it and press it forwards towards the base of the tongue. In some individuals the finger may be made to overlap the epiglottis to the extent of three-fourths of an inch.

Thus placed, the finger serves as a sure guide to the instrument to be used. The knife is then to be conducted with its concavity directed downwards, along the finger till its point reaches the finger nail. By elevating the handle so as to depress the blade an inch to an inch and a half further, the cutting extremity is placed in the glottis between its edges; at this stage of the operation the knife is to be slightly rotated to one side and the other, giving it a cutting motion in the act of withdrawing it. This may be repeated without removing the finger two or three times on either side. The margin of the epiglottis, and the swelling between it and the base of the tongue may be scarified still more easily with the same instrument, or scissors curved flatwise may be employed for these parts, guided in the same manner as the knife.

Though a disagreeable sense of suffocation and choking is caused by the operation, the patient soon recovers from it, and submits to a repetition after a short interval. In every instance the operation has been performed twice, and in some three times.

Before proceeding to the operation, it has always been explained to the patient that the seat of his difficulty was a swelling at the top of the windpipe, preventing the air from entering, and the object of the operation was to cut it and let out fluid, and thus give him relief. This explanation corresponds so exactly with his own sensations, which refer to the top of the thyroid cartilage as the seat of obstruction, that he readily submits to the proposed operation, and renders all the co-operation in his power for its performance.

A slight hemorrhage follows the scarifications, and should be encouraged by gargling with warm water. In one instance the quantity of blood mixed with sputa amounted to half a wineglassful.

Dr. Buck's first operation was performed on the 13th of April, 1847. The patient was a seaman, thirty-one years old. After exploring the parts with the finger, and ascertaining the existence of the swelling of the epiglottis, and also allowing his two assistants to do the same, he scarified the aryteno-epiglottic folds and the epiglottis, partly with scissors curved flatwise, and partly with a sharp pointed bistoury, guarded to within one-third of an inch of its point by a narrow strip of adhesive plaster wound around it, and conducted to the parts upon the fore-finger of the left hand, previously introduced at the right angle of the mouth. Two or three repetitions were requisite, at short intervals, to complete the operation. The patient hawked up three or four teaspoonsful of blood, mixed with mucus, and expressed himself as feeling relieved. He was then bled to the extent of twenty ounces, and took grain doses of tartar emetic. He continued to improve daily, and was discharged well, on the 23d.

Dr. Buck's second case was operated upon, January 13th, 1848. The patient was thirty years old, and was attacked with the disease the day before. In addition to the ordinary well marked symptoms of the disease, the epiglottis was distinctly felt to be swollen, and its margin thickened and folded together, both by Dr. Buck and Dr Swett. The pouches between the epiglottis and the base of the tongue were filled up by a soft pulpy swelling. At 10½ o'clock, Dr. Buck scarified the edges of the glottis as well as the epiglottis, and the swelling anterior to it, with the bistoury and curved scissors, as in the first case. Slight hemorrhage followed, and was encouraged by a warm water gargle. The exploration of the parts, as well as the operation itself, did not cause much disturbance, and the patient expressed decided relief. At 2½ o'clock, P. M., the same day, he breathed more calmly and felt still further relief. He passed the following night very comfortably, and the next morning expressed himself quite well. The swollen parts were ascertained by the touch to have very much diminished. He recovered without any repetition of the scarifications.

The third operation was done on the 29th of February. The patient was 50 years old. The epiglottis was examined by the touch, by Dr. Buck, and by several of his colleagues, and felt to be swollen, thickened, especially at its margin, pulpy and convoluted upon itself. The patient had been bled, vomited, and had taken twenty grains of calomel. The edges of the glottis and epiglottis were scarified with a curved instrument, designed by Dr. Buck expressly for this purpose, the operation being repeated two or three times, at a few moments' interval. The patient was relieved and continued comfortable till the second day, when the dyspnea having increased, the scarifications were repeated with the guarded bistoury, but the operation was not satisfactorily done. On the third day the patient was worse, and the epiglottis was felt to be more swollen. At 3 o'clock, P. M., the scarifications were repeated with the curved knife. At 6 o'clock, P. M., although the patient expressrd himself as somewhat relieved, the dyspnea was so urgent that Dr. Buck and Dr. Hoffman urged him to submit at once to tracheotomy. This he refused to do. An erysipelatous blush had shown itself on the right cheek. On the following day the patient was rather better, and then steadily improved till he was well. He took calomel and antimony regularly during the continuance of the disease.

The third case occurred nearly at the same time and was cured by the same means. There was nothing special in its history to render it worth while to give an abstract of it.

The fifth operation was performed on the 5th of March. The patient was an athletic seaman, 24 years old. " He lay," says Dr. Buck, " upon his right side, with his face near the edge of the bed, his eyes closed, and his countenance pale and of a leaden hue. His features were altered and of an almost death-like expression; the skin was bathed in perspiration, and every muscle of the trunk seemed to be brought into powerful action to perform the act of inspiration, which was protracted and sonorous, while that of expiration was short, easy, and unobstructed. This condition had existed about six hours." The evening previous, Dr. Swett had ascertained by the touch the existence of a moderate degree of swelling of the epiglottis. No time was lost in scarifying the edges of the glottis and epiglottis, with the curved knife. The operation was repeated two or three times at short intervals, and followed by a small quantity of blood in the sputa. After waiting half an hour, it was thought by Dr. Buck and Dr. Swett, most prudent, considering the urgency of the symptoms, not to rely exclusively upon the scarifications, but to give the patient the additional chance of tracheotomy. This was accordingly performed without delay. Great difficulty was encountered from the depth of the trachea, the swelling of the superjacent parts, the resistance of the patient, and the copious venous hemorrhage. On incising perpendicularly the three superior rings of the trachea, the air rushed in with great force, and breathing was soon established through the artificial passage. The happiest effects promptly followed. The patient, from being exceedingly turbulent and excited, became tranquil and submissive. The respiration grew calm and required no effort, and the countenance resumed its natural expression. On the following day the patient was doing well and the swelling of the epiglottis had decidedly diminished. After removing the tube from the trachea, to cleanse it, the sides of the wound were pressed together so as to close the opening, when it was found that respiration through the larynx could be performed with considerable facility, showing clearly that the swelling of the glottis had already very much diminished. On the second day the patient was able to breathe with natural facility through the larynx. He recovered rapidly and entirely.*

Such is a brief abstract, given in good part in his own words, of Dr. Buck's five cases.

They constitute, it seems to me, one of the most interesting and brilliant series of cases to be found in the records of medical art. It is very true that the operation, as has already been stated, was not a new one, and that it had been several times successfully performed many years ago. But it is also true that it had not only failed to attract any general attention, and to establish itself in

*Trans. Amer. Med. Asso. vol. i, pp. 137, 144.

the confidence of the medical public, but it had been doubtingly and disparagingly spoken of by one, at least, of the most distinguished and authoritative contemporaries of its author. Dr. Buck was not aware that the operation had ever before been either performed or proposed. It may perhaps turn out, hereafter, that these five successive and successful cases have been marked by that unwonted favorable fortune which seems to accompany, so frequently, the advent and the early history of new remedies and new methods of treatment, and that occasional or frequent failures may occur to shake the confidence which these cases have created in the operation, and to lower in a corresponding degree our estimate of its value; but there is every reason, I think, that can be derived from the nature of the disease, and of the operation, not only to hope but to believe that such wi l not be the case; and that this simple, direct, and rational method of cure, first devised and performed by Lisfranc, and after the neglect if not the general forgetfulness of quarter of a century, again originated and revived by Dr. Buck, will take its fixed and permanent place amongst the established and most valuable resources of art. One of my principal objects for making this compilation was to aid in diffusing an adequate knowledge of this operation.

The choice of an instrument for the performance of this operation is a matter of some importance. Lisfranc used a long narrow-bladed slightly curved bistoury in a stiff handle, protected with a strip of linen, to within half an inch of the point. Dr. Buck, in his two first cases, made use of a sharp pointed curved bistoury, guarded to within one-third of an inch of its point by a narrow strip of adhesive plaster wound around it, and of scissors curved flatwise. He then had a curved knife designed expressly for the operation. Its general form is very much like that of the common gum lancet, with the fixed handle, used by dentists. It is about an inch longer, and the curve, instead of being half an inch or so in length, as in the lancet, is about an inch long. It is not probable that practitioners will generally provide themselves with Dr. Buck's knife. It is important for them to know that the curved bistoury properly guarded, or the dentist's gum lancet, will answer the purpose. For want of a better, and in case of emergency, an instrument might be made by fastening, simply by winding thread around them, the blade of a common gum lancet to a straight stiff handle, of suitable length. Let it be remembered that the operation is never to be omitted or deferred for want of exactly proper or suitable instruments, since it can be done with such as are of ruder contrivance and less adapted to the purpose even than any above mentioned. In any event the physician has his index fingers; let him roughen and sharpen the nails of these, and he may thus lacerate and tear the infiltrated tissues so as to give exit to their contents.

SEC. 3. *Tracheotomy.*—Setting aside, for the present, the preceding operation, or supposing it to be for any reason either impracticable or unsuccessful, there can be no doubt whatever, of the great value and the paramount importance of tracheotomy. In case of either of the above contingencies, it ought undoubtedly to be resorted to, and it ought to be performed, always, before the phenomena of asphyxia have become much developed. The chief cause of its frequent failure is without doubt to be found in its having been too long delayed.

### ARTICLE XI.—DEFINITION.

*Edematous laryngitis, or edema of the glottis*, is a serous purulent, or sero-purulent infiltration of the lax submucous cellular tissue of the glottis and its immediate neighborhood; affecting especially the aryteno-epiglottic folds, the upper surface and the borders of the epiglottis, rendering the tissue tense and tumid; accompanied in most cases with a pale, whitish, red, or blueish red color, or with some other morbid condition of the mucous membrane covering the infiltrated parts; sometimes occurring suddenly, but in most cases coming on somewhat gradually, and marked by the following symptoms, to wit:—pain, or uneasiness, more or less distressing, at the upper part of the larynx; a sensation of a foreign body lodged in the posterior fauces; cough; hoarseness, feebleness, or loss of voice; in many cases, more or less difficulty of deglutition; in all cases, difficult and labori ous respiration, the inspiration usually much more so than the expiration; the dyspnea constant, but attended by occasional paroxysms of terrible and frightful intensity, and steadily increasing in degree and severity, unless relieved by art; the infiltrated and tumefied parts capable of being reached and their condition appreciated by the finger: the march of the disease rapid; its duration short, varying from a few hours to a few days; occurring, in nearly all cases, in subjects already suffering with disease, and especially in patients convalescing from fevers of a typhoid character; and in persons already affected with some local inflammation in the vicinity of the glottis; terminating almost constantly in death, unless relieved by art, only to a very moderate extent under the control of medicine, but susceptible of entire relief, in most cases, by scarification of the infiltrated tissue, and the consequent escape of the effused fluid, aided, where there is a pressing necessity for it, by tracheotomy.

### ARTICLE XII.—History and Bibliography.

The first historian of edematous laryngitis, in any degree complete and authentic, was Dr. G. L. Bayle, of Paris. The disease is his, by the same title that albuminuria is Dr. Bright's. His original Memoir on Edema of the Glottis, or Edematous Laryngeal Angina, was read before the Society of the School of Medicine, of Paris, on the

18th of August, 1808. This memoir was not published till 1819, when it appeared in the Journal de Medecine et de Chirurgie. The first publication by Bayle, appears to have consisted of the substance of this memoir, as an article in the Dictionary des Sciences Medicales, in 1817. This article contains a very clear, succinct, and accurate account of the disease, to which, subsequent and more varied and extensive observations have made but few additions.

Several of the older anatomists and pathologists, amongst whom may be mentioned Bonetus, Morgagni, and Lieutaud, had seen and described the edematous condition of the glottis, and were aware of its formidable nature, but they left no full and explicit account of the disease. Bichat had, evidently, a very correct knowledge of the disease. In his Descriptive Anatomy, published before the appearance of Bayle's Memoir, he says: "It is certain that the portion of the laryngeal mucous membrane which forms the superior opening of the larynx, is subject to a peculiar species of serous engorgement, which does not manifest itself in any other place, and which, thickening very greatly the walls, suffocates the subjects in a very short time." In another place, after giving some account of experiments upon dogs, in which he made incisions below the tongue and pierced the epiglottis, he says: "In one instance, a dog, submitted to this experiment, died the following day, with a serous angina, exactly like that with which patients are all at once suffocated, and which has its seat in the two folds of mucous membrane extending from the arytenoid cartilages to the epiglottis."*

The Essay of Tuilier was published as a thesis in 1815. It was important and valuable principally from its containing an account of the signs of the disease, derived from the touch. Bayle says of it: "There will be found

*Mem. de l'Acad. Roy. de Med., vol. xi., p. 88.

in this essay—1st. A report of four cases which had not before been published. 2nd. An account of the pathognomonic palpable sign of the disease. And 3d. Various points of doctrine very ably discussed, especially that relative to the introduction of a sound into the larynx, proposed in 1813, by M. Louis-Benoit Finaz de Seizel.—I have not seen this thesis of Tuilier; and I derive my knowledge of it from Bayle and Valleix.

Lisfranc's memoir was published in the Journal Generale for 1825. Cruveilhier's article, in which he expresses his disbelief in the fact of Lisfranc's operation ever having been performed, is contained in the eleventh volume of the Practical Dictionary of Medicine and Surgery, published in 1834. Trousseau and Belloe's prize essay upon laryngeal phthisis, containing important observations upon the disease, was published in 1837. Legroux recommended lacerating the swollen parts with the finger nail, in 1839.*

The full and elaborate memoir, by Valleix, is contained in the eleventh volume of the Memoirs of the Royal Academy of Medicine. The occasion of its being written was a question proposed by the Academy in the following terms: "*To ascertain what are the causes of edematous laryngeal angina (edema of the glottis); to make known its march, its successive symptoms, and its differential diagnosis; to discuss, in its treatment, the advantages and disadvantages of tracheotomy.*" The memoir occupies one hundred and ten pages, in quarto, of the volume, and, it is hardly necessary to say, is marked throughout by the clearness, ability, and sound practical philosophy of its author, and of the school of observation to which he belongs.

Dr. Buck's paper is contained in the first volume of the Transactions of the American Medical Association.

---

*Valleix. Mem. de l'Acad. Roy. de Med., vol. xi., p. 89.

